# Supplementary material for: Deep Networks Can Resemble Human Feed-forward Vision in Invariant Object Recognition
Source: Sci Rep. 2016 Sep 7;6:32672. doi: 10.1038/srep32672 (PMC5013454; doi:10.1038/srep32672)
Supplement: Supplementary Information [file srep32672-s1.pdf]

Deep Networks Can Resemble Human Feed-forward Vision in Invariant Object Recognition

Saeed Reza Kheradpisheh[1,6], Masoud Ghodrati[2], Mohammad Ganjtabesh[1,*], and Timothée Masquelier[3,4,5,6,*]

[1]Department of Computer Science, School of Mathematics, Statistics, and Computer Science, University of Tehran, Tehran, Iran
[2]Department of Physiology, Monash University, Melbourne, VIC, Australia
[3]INSERM, U968, Paris, F-75012, France
[4]Sorbonne Universités, UPMC Univ Paris 06, UMR-S 968, Institut de la Vision, Paris, F-75012, France
[5]CNRS, UMR-7210, Paris, F-75012, France
[6]CERCO UMR 5549, CNRS – Université de Toulouse, F-31300, France
[*]Corresponding authors, e-mails: mgtabesh@ut.ac.ir, timothee.masquelier@alum.mit.edu

Supplementary information

Confusion matrices

In addition to the last layer of each model, we have also computed the confusion matrices of all layers of DCNN models across different variation levels. For all variation levels and both natural and plain background conditions, confusion matrices of different layers of Krizhevsky et. al. 2012, CNN-F 2014, CNN-M 2014, CNN-S 2014, OverFeat 2014, Hybrid-CNN 2014, and Zeiler and Fergus 2013 are presented in Fig. S1 to S7, respectively. Furthermore, the confusion matrices of HMAX, Pixel and humans are provided in Fig. S8 to S10, respectively. In general, by increasing the level of variations the recognition error rate also increases, faster in lowest layers and more slowly in highest ones. As mentioned in the paper, this indicates that invariance gradually increases in DCNNs and culminates in the highest layers.

Another important fact to mention is the error distributions of DCNNs across the levels and layers. It is evident that the major portion of miss-classified samples in Krizhevsky et. al. 2012, Zeiler and Fergus 2013, and Hybrid-CNN, for all layers and variation levels, belong to the car and motorcycle classes. On the other side, for all layers and variation levels, the animal and airplane classes have the highest miss-classification rates in CNN-F, CNN-M and CNN-S models. Hence, it can be concluded that inappropriate object representations in low layers deeply affect object representation and recognition in the highest layers. Of course, the weakness of lower layers in DCNNs stems in the top down nature of the gradient descent learning (back-propagation) algorithm which projects the errors of top layers into the layer below. On the other hand, unsupervised learning algorithms mainly focus on image-driven features which frequently appeared in input images regardless of their categorical information. Therefore, a combination of unsupervised image-based learning and supervised error-based learning algorithms which cover the weak-points and amplifying the strength of both approaches might be more successful.

Representational dissimilarity matrices (RDMs)

The RDMs of the last layers of DCNNs as well as humans, HMAX and Pixel model are presented in the main text. Here, we provide the RDMs of all layers of DCNNs (see Fig. S11 to S17), along with the RDMs of HMAX, Pixel, and humans (see Fig. S18 to S20) for different variation levels in both natural and plain background conditions. In addition, the RDMs of layers 9 to 18 of Very Deep model over different levels of the natural background task are provided in Fig. S21. Such illustrations help us to track how the measure of invariance progressively increases across the layers of DCNNs for simple, intermediate and difficult variation levels. Overall, in each variation level, by moving from lower to higher layers the within-class dissimilarities increase while between-class dissimilarities are decreasing. At the same time for a specific layer, by increasing the variation level the within- and between-class dissimilarities decrease and increase, respectively. In sum, one can conclude that invariant object representation continuously improves across the layers of DCNNs, but even the highest layers are not sufficiently robust for broad object variations. In addition, for all variation levels, considering the RDM and confusion matrix of each layer of each DCNN model indicates that those categories which are not well represented have the highest miss-classification rates.

Separability index analysis

In another experiment, we evaluated the representational geometry of the models in term of categorical similarity. We computed the category separability index for the internal representations of each model by computing the ratio of within-category relative to between-category dissimilarities. We first computed the mean within-category dissimilarity by averaging the elements of the RDM corresponding to the same category images. We also computed the mean between-category dissimilarity in a

similar manner but using the other elements of the RDM. Then, we divided the mean within-category dissimilarity by the mean between-category dissimilarity and subtracted it from one. Hence, a model with lower within-category and higher between-category dissimilarities would gain a higher separability index.

Figure S22 shows the separability index for different layers of all models for all variation levels and background conditions. Comparing Fig. S22A and Fig. S22B reveals that adding natural backgrounds reduces the separability index of models by a factor ~ 2, indicating that cluttered background can disturb the categorical similarity. Also, the separability index increases in higher layers and culminates in the final fully connected layers. By juxtaposing the results of performance (see Fig. 3 and Fig. 4 of the main manuscript) and separability index (Fig. S22) analysis, it can be concluded that a model with a lower separability index might nevertheless have a higher accuracy than a model with a higher separability index. For instance, the fully connected layers of DCNNs in natural background experiment have lower accuracies than the last convolutional layers, while they have higher separability indexes. Also, CNN-F, CNN-M and CNN-S have rather low separability indexes but the best accuracies. In fact, it is the actual positions of images of different categories in the representational space which determines the final accuracy of a model, not just the mean inter- and intra-class distances.

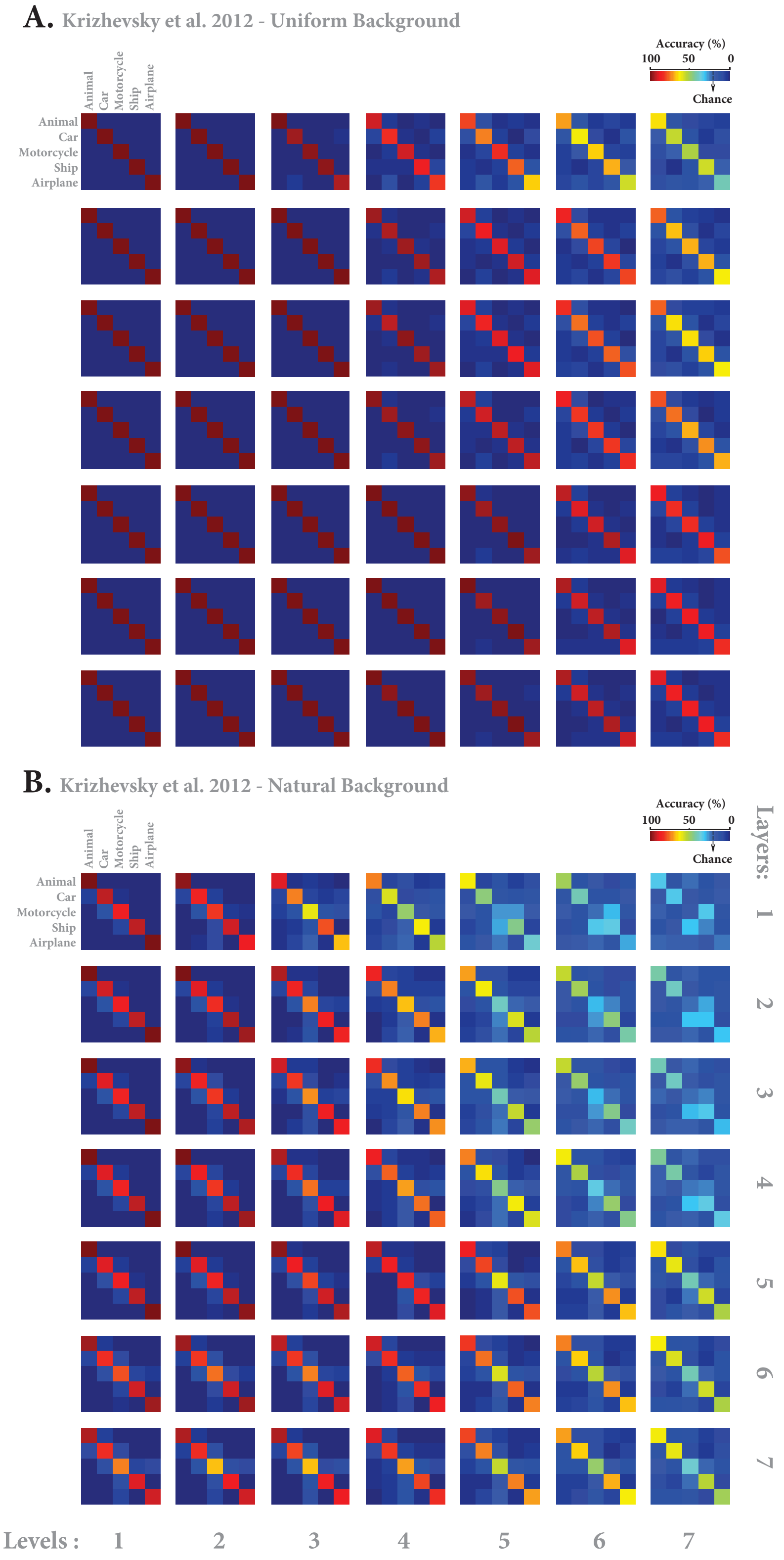

Figure S1. Confusion matrices of different layers of Krizhevsky et. al. 2012 for multiclass invariant object categorization tasks in A) plain and B) natural background conditions. Each row corresponds to a layer and each column shows a particular level of variation (level 1-7). Each color-coded matrix shows the accuracy of a model in categorizing different object categories (specified in the first matrix at the top-left corner). The color bar at the top-right shows the percentage of the labels assigned to each category. The chance level is specified with an arrow on the color bar.

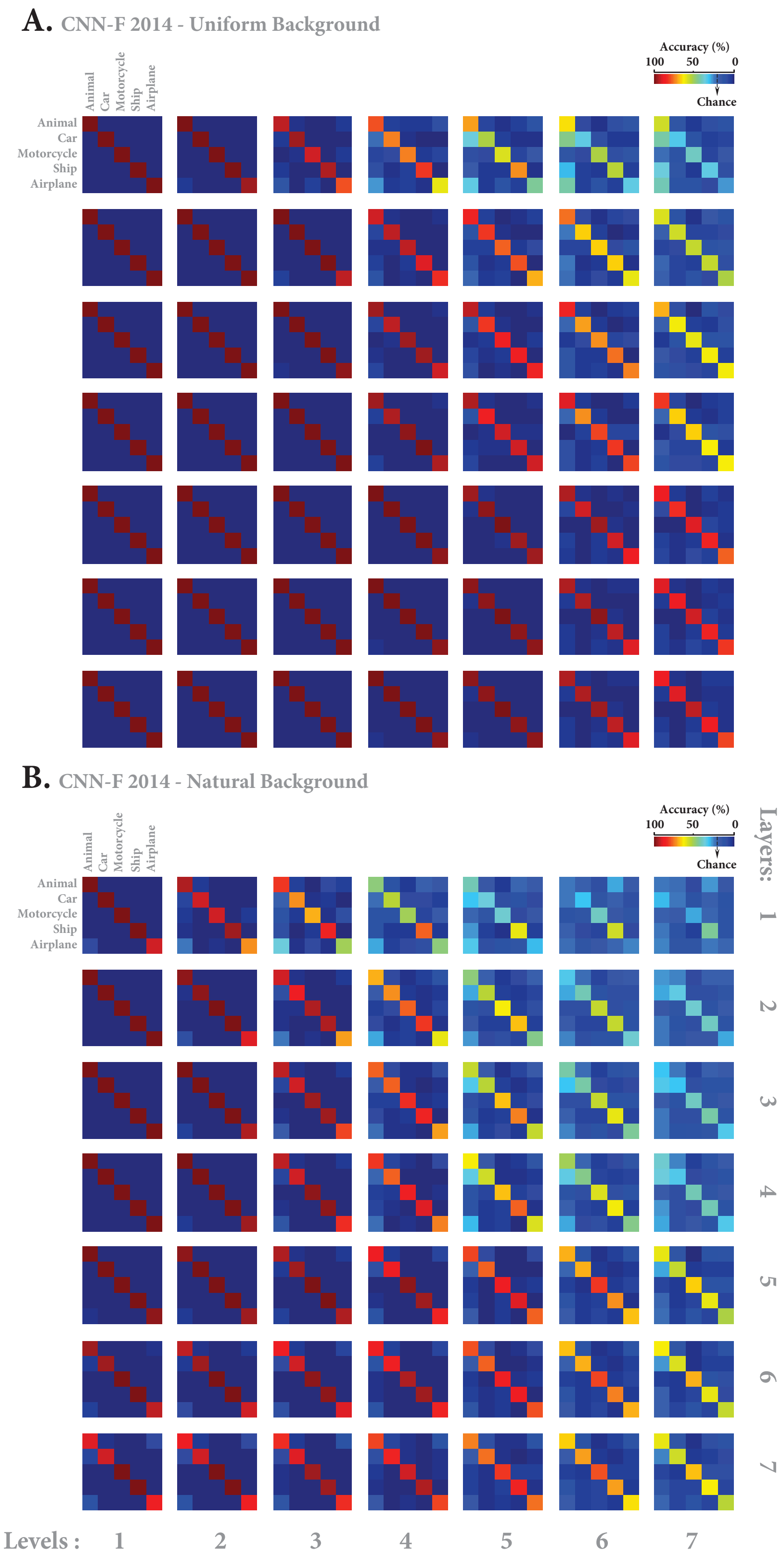

Figure S2. Confusion matrices of different layers of CNN-F 2014 for multiclass invariant object categorization tasks in A) plain and B) natural background conditions. Each row corresponds to a layer and each column shows a particular level of variation (level 1-7). Each color-coded matrix shows the accuracy of a model in categorizing different object categories (specified in the first matrix at the top-left corner). The color bar at the top-right shows the percentage of the labels assigned to each category. The chance level is specified with an arrow on the color bar.

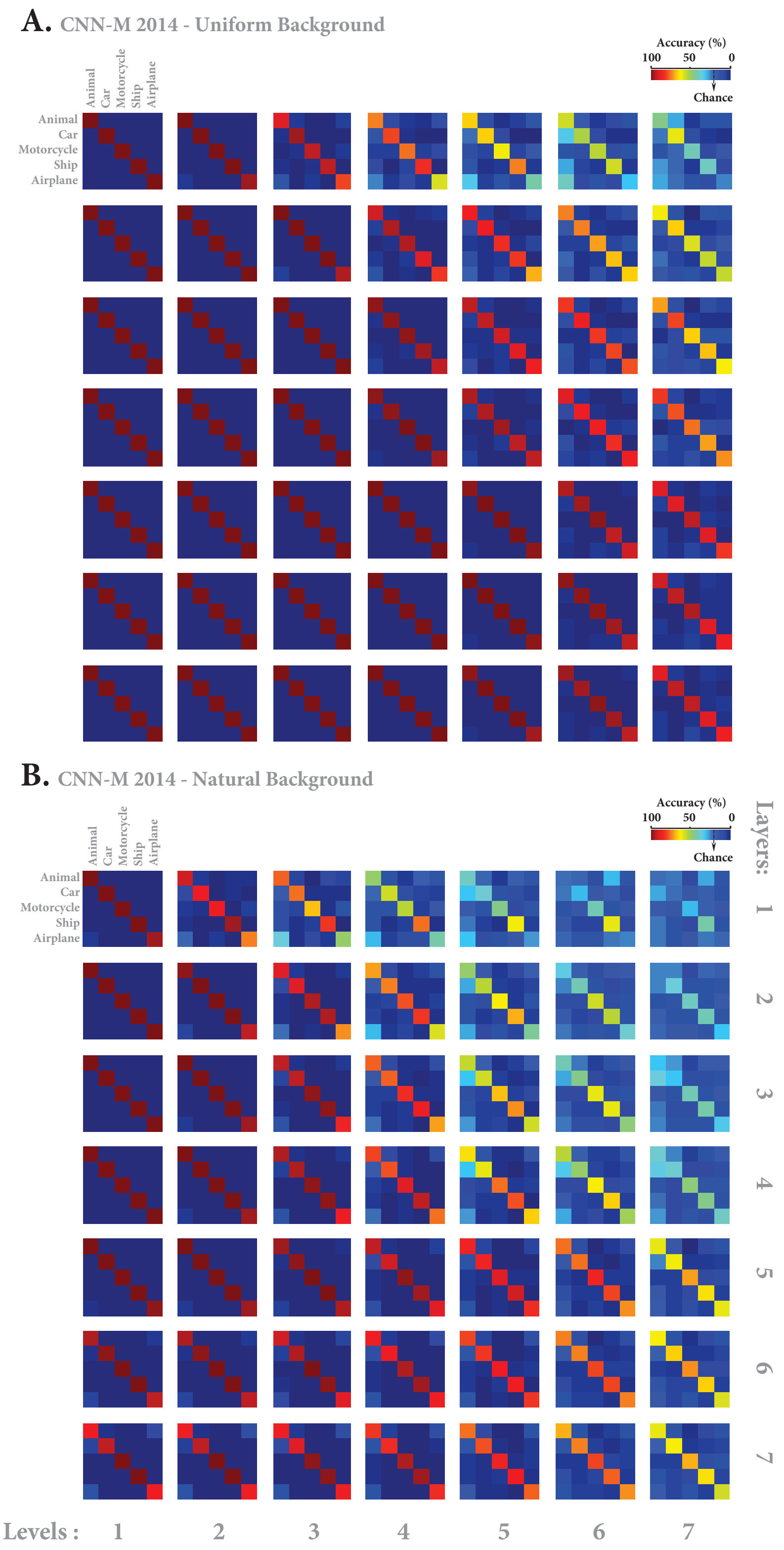

Figure S3. Confusion matrices of different layers of CNN-M 2014 for multiclass invariant object categorization tasks in A) plain and B) natural background conditions. Each row corresponds to a layer and each column shows a particular level of variation (level 1-7). Each color-coded matrix shows the accuracy of a model in categorizing different object categories (specified in the first matrix at the top-left corner). The color bar at the top-right shows the percentage of the labels assigned to each category. The chance level is specified with an arrow on the color bar.

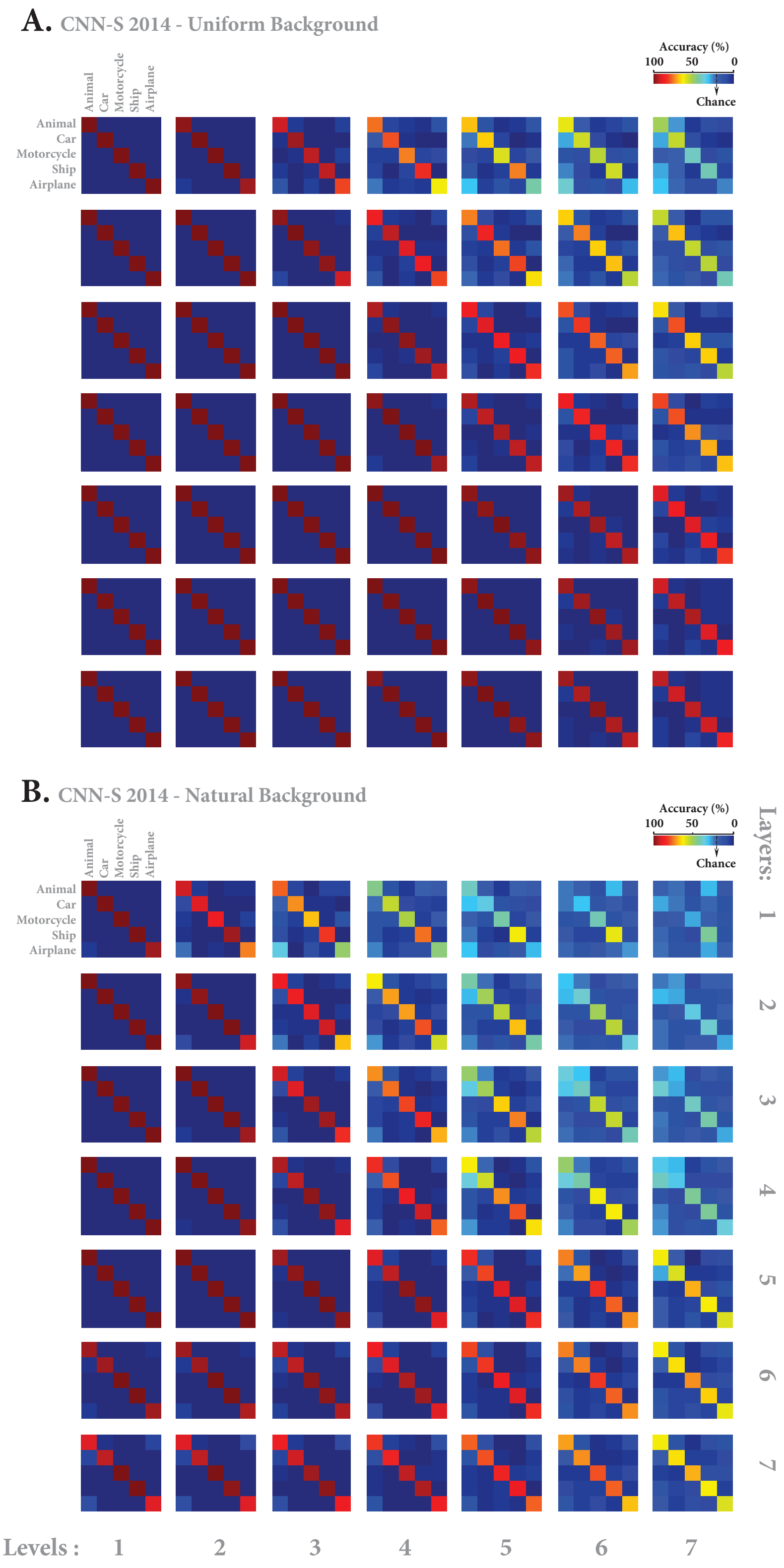

Figure S4. Confusion matrices of different layers of CNN-S 2014 for multiclass invariant object categorization tasks in A) plain and B) natural background conditions. Each row corresponds to a layer and each column shows a particular level of variation (level 1-7). Each color-coded matrix shows the accuracy of a model in categorizing different object categories (specified in the first matrix at the top-left corner). The color bar at the top-right shows the percentage of the labels assigned to each category. The chance level is specified with an arrow on the color bar.

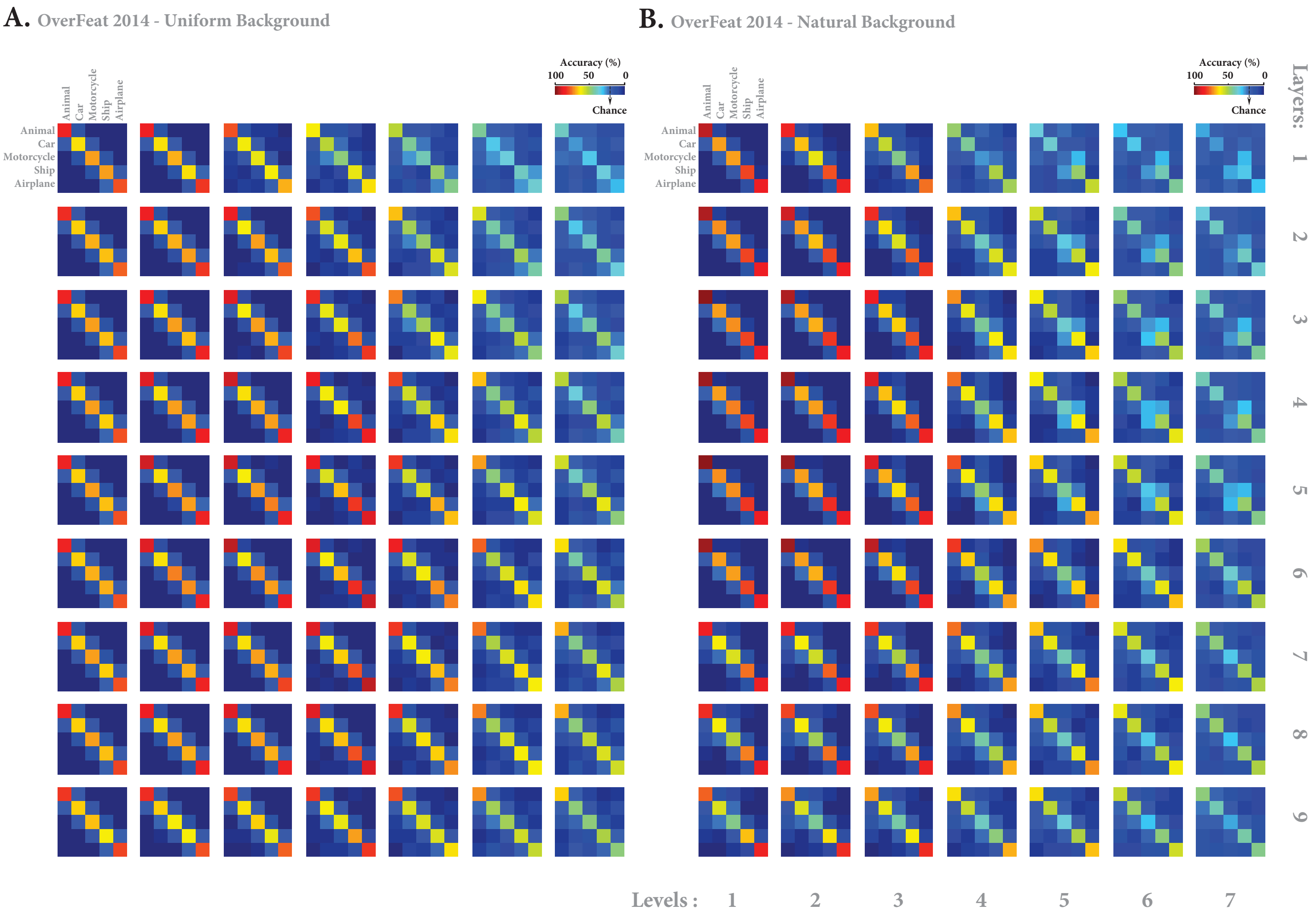

Figure S5. Confusion matrices of different layers of OverFeat 2014 for multiclass invariant object categorization tasks in A) plain and B) natural background conditions. Each row corresponds to a layer and each column shows a particular level of variation (level 1-7). Each color-coded matrix shows the accuracy of a model in categorizing different object categories (specified in the first matrix at the top-left corner). The color bar at the top-right shows the percentage of the labels assigned to each category. The chance level is specified with an arrow on the color bar.

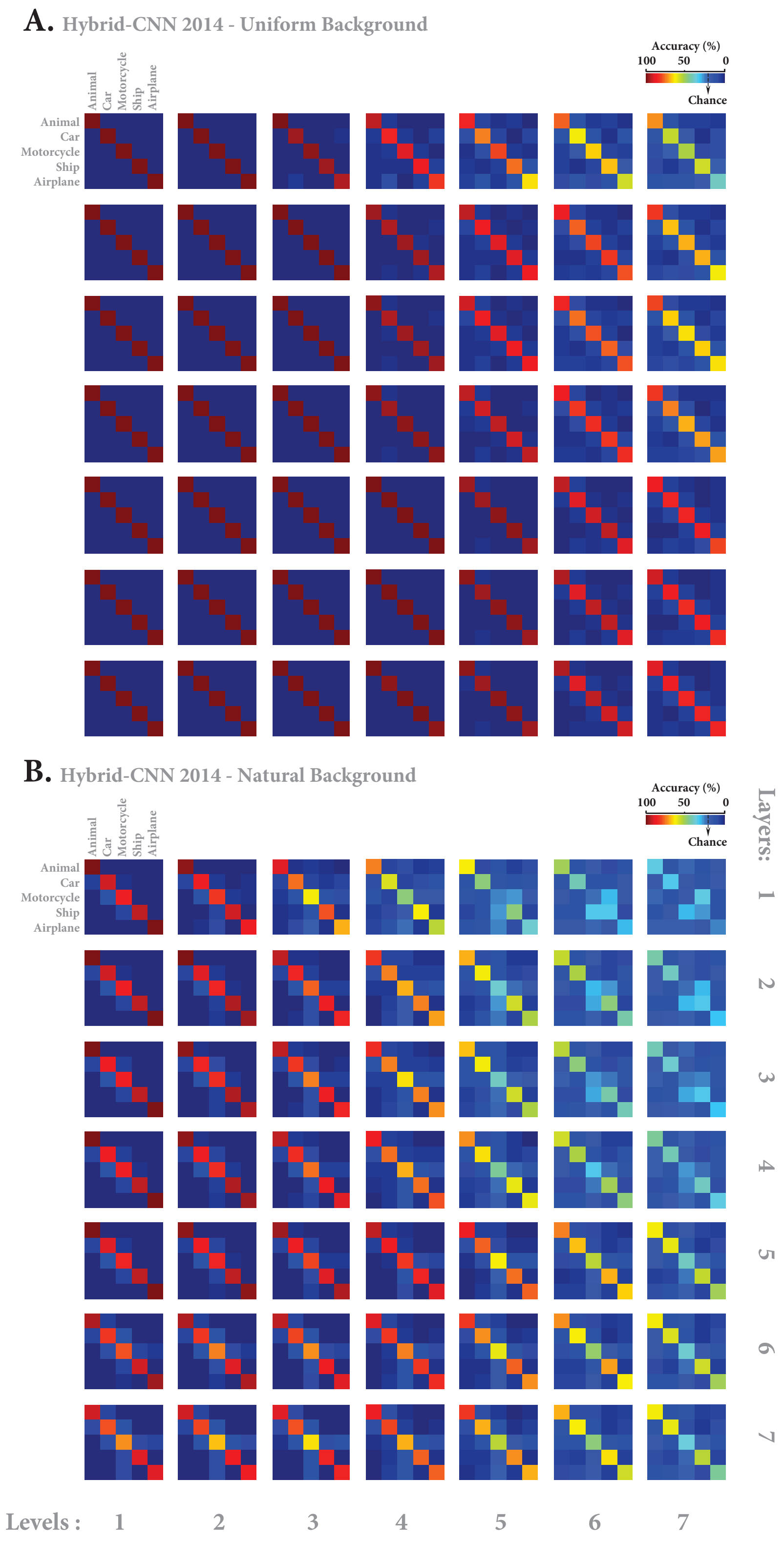

Figure S6. Confusion matrices of different layers of Hybrid-CNN 2014 for multiclass invariant object categorization tasks in A) plain and B) natural background conditions. Each row corresponds to a layer and each column shows a particular level of variation (level 1-7). Each color-coded matrix shows the accuracy of a model in categorizing different object categories (specified in the first matrix at the top-left corner). The color bar at the top-right shows the percentage of the labels assigned to each category. The chance level is specified with an arrow on the color bar.

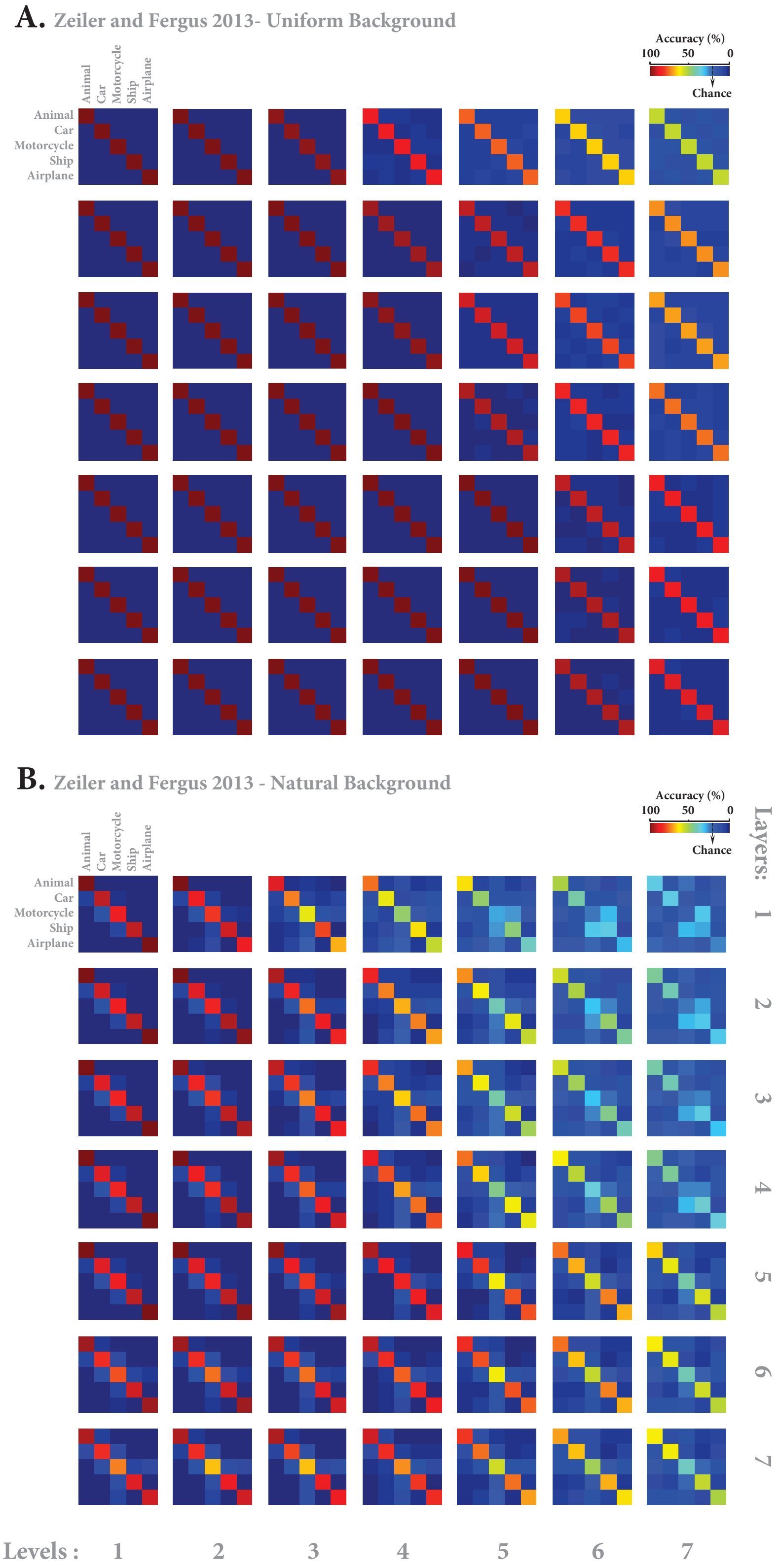

Figure S7. Confusion matrices of different layers of Zeiler and Fergus 2013 for multiclass invariant object categorizations task in A) plain and B) natural background conditions. Each row corresponds to a layer and each column shows a particular level of variation (level 1-7). Each color-coded matrix shows the accuracy of a model in categorizing different object categories (specified in the first matrix at the top-left corner). The color bar at the top-right shows the percentage of the labels assigned to each category. The chance level is specified with an arrow on the color bar.

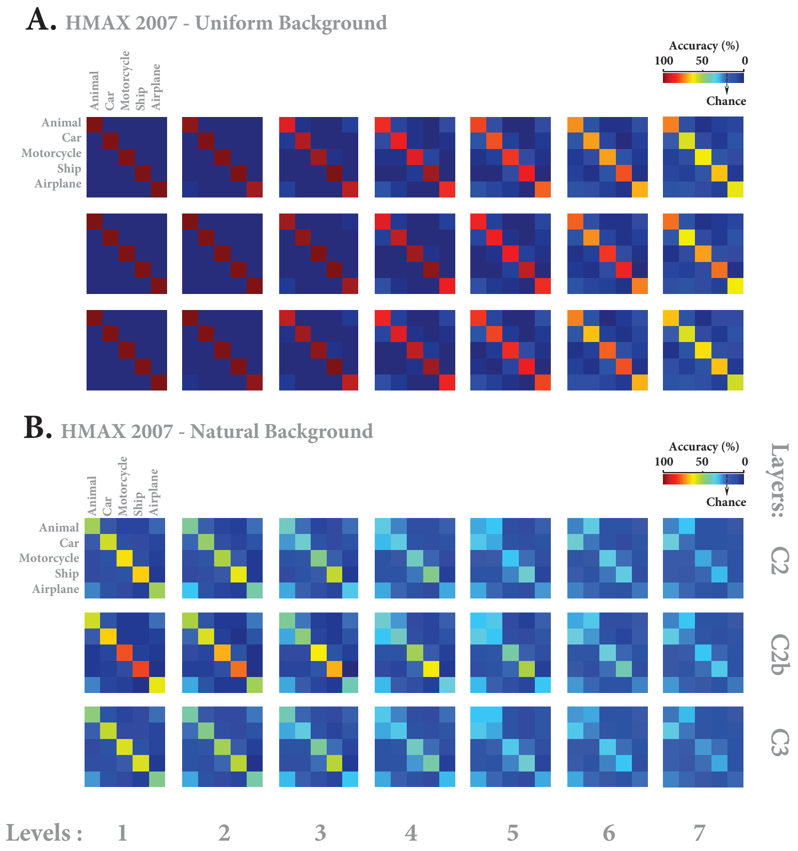

Figure S8. Confusion matrices of different layers of HMAX for multiclass invariant object categorization tasks in A) plain and B) natural background conditions. Each row corresponds to a layer and each column shows a particular level of variation (level 1-7). Each color-coded matrix shows the accuracy of a model in categorizing different object categories (specified in the first matrix at the top-left corner). The color bar at the top-right shows the percentage of the labels assigned to each category. The chance level is specified with an arrow on the color bar.

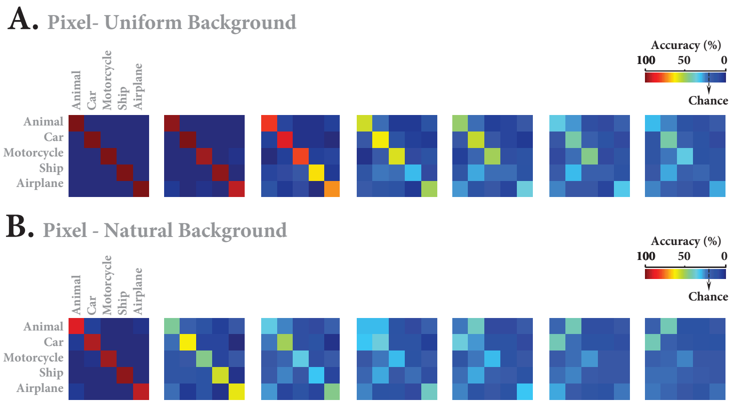

Figure S9. Confusion matrices of Pixel model for multiclass invariant object categorization tasks in A) plain and B) natural background conditions. Each color-coded matrix corresponds to a particular level of variation (level 1-7) and shows the accuracy of a model in categorizing different object categories (specified in the first matrix at the top-left corner). The color bar at the top-right shows the percentage of the labels assigned to each category. The chance level is specified with an arrow on the color bar.

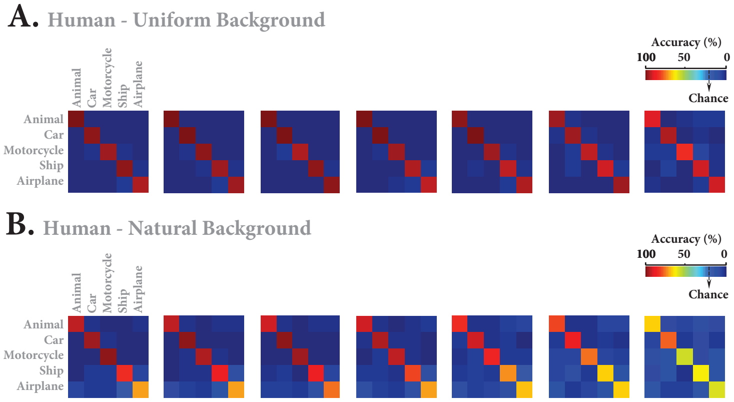

Figure S10. Confusion matrices of humans for multiclass invariant object categorization tasks in A) plain and B) natural background conditions. Each color-coded matrix corresponds to a particular level of variation (level 1-7) and shows the accuracy of a model in categorizing different object categories (specified in the first matrix at the top-left corner). The color bar at the top-right shows the percentage of the labels assigned to each category. The chance level is specified with an arrow on the color bar.

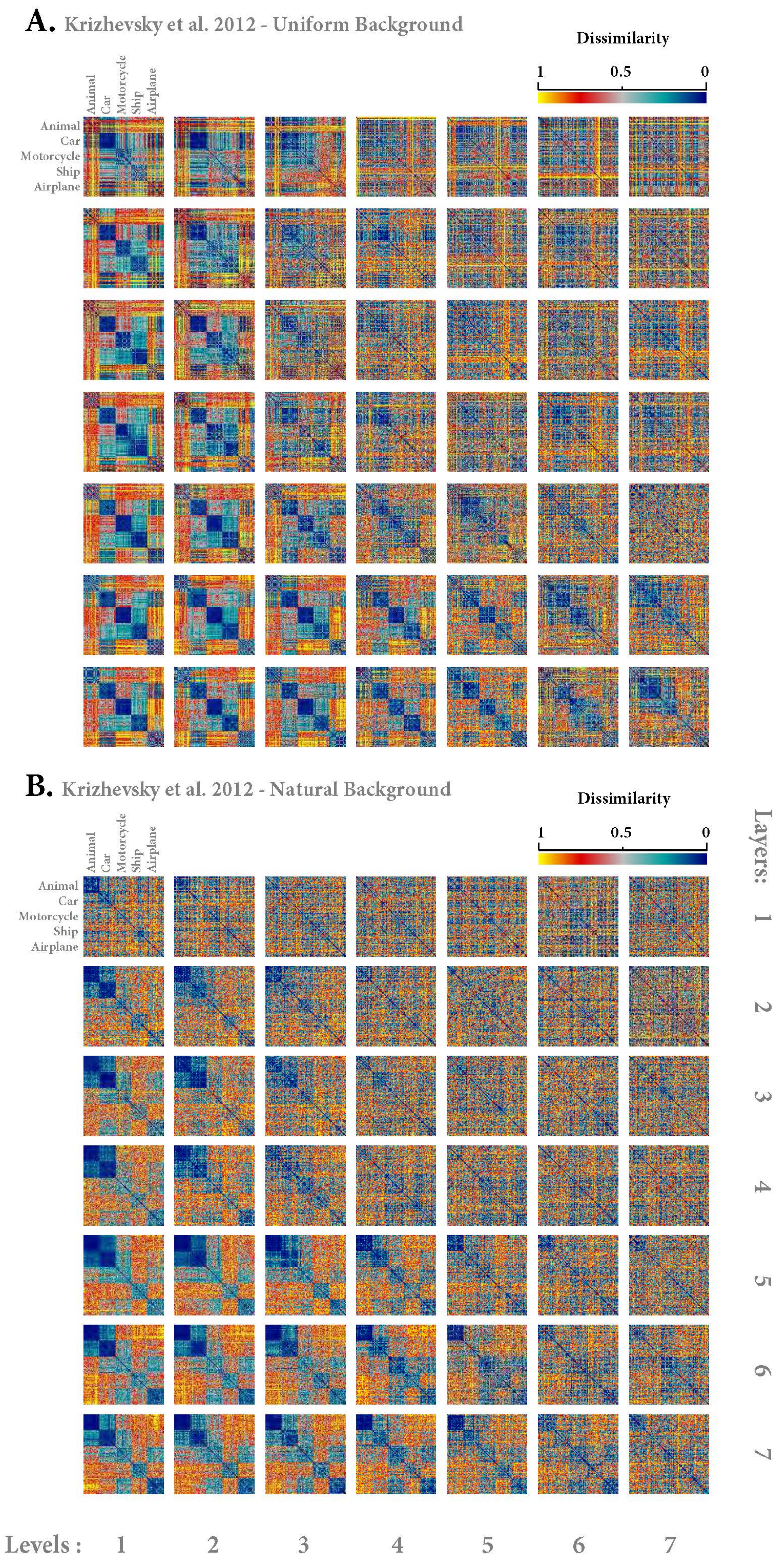

Figure S11. Representational Dissimilarity Matrices (RDM) for different layers of Krizhevsky et. al. 2012 in A) plain and B) natural backgrounds. Each element in a matrix shows the pairwise dissimilarities between the internal representations of a model for two images (measured as 1-r, Spearman's rank correlation. See Materials and Methods). Each row of RDMs corresponds to a layer and each column shows a particular level of variation (from level 1-7). The color bar at the top-right corner shows the degree of dissimilarity. The size of each matrix is 100×100, with 20 randomly selected images from each category. This was done for the sake of better visualization.

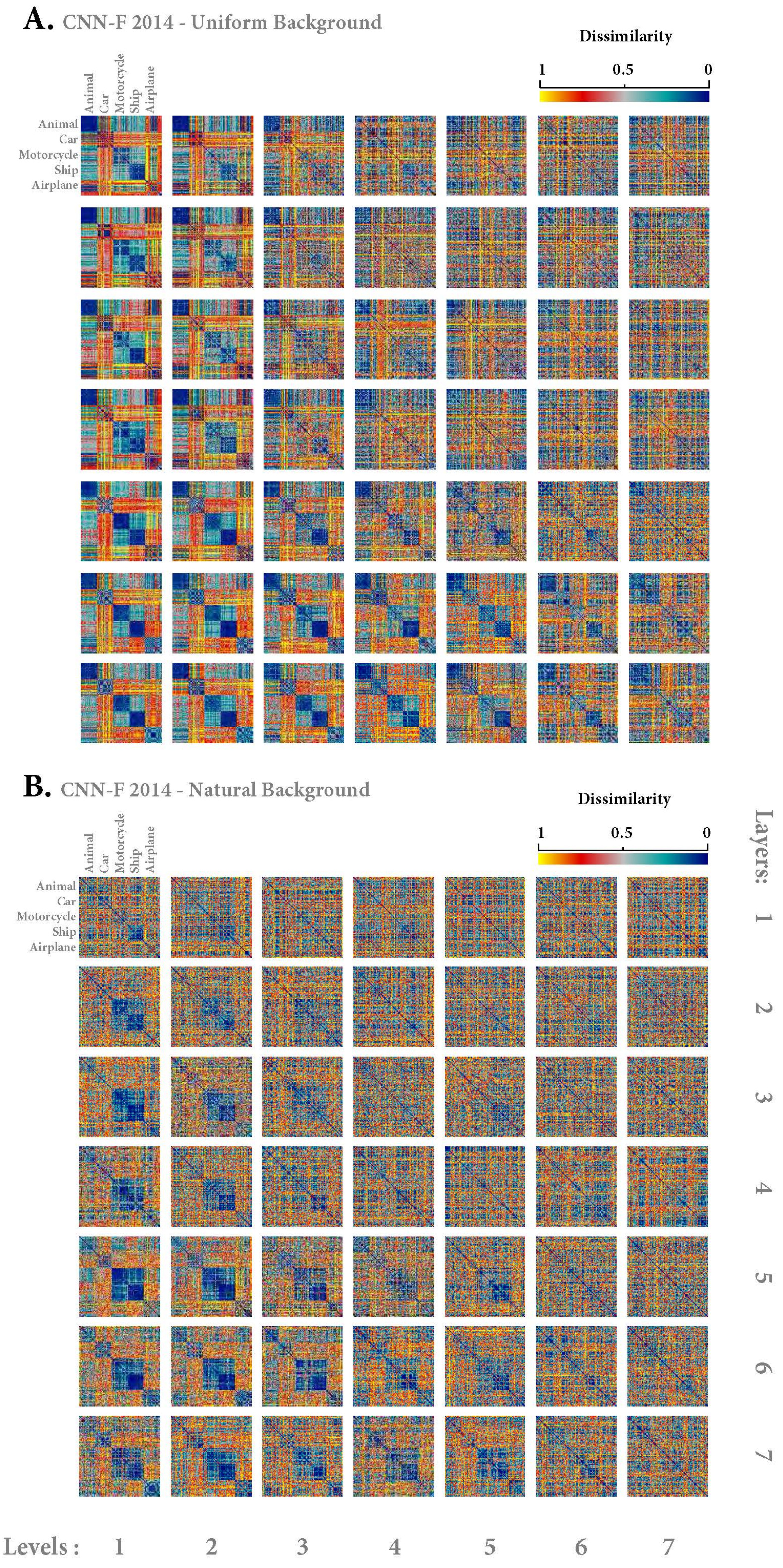

Figure S12. Representational Dissimilarity Matrices (RDM) for different layers of CNN-F 2014 in A) plain and B) natural backgrounds. Each element in a matrix shows the pairwise dissimilarities between the internal representations of a model for two images (measured as 1-r, Spearman's rank correlation. See Materials and Methods). Each row of RDMs corresponds to a layer and each column shows a particular level of variation (from level 1-7). The color bar at the top-right corner shows the degree of dissimilarity. The size of each matrix is 100×100, with 20 randomly selected images from each category. This was done for the sake of better visualization.

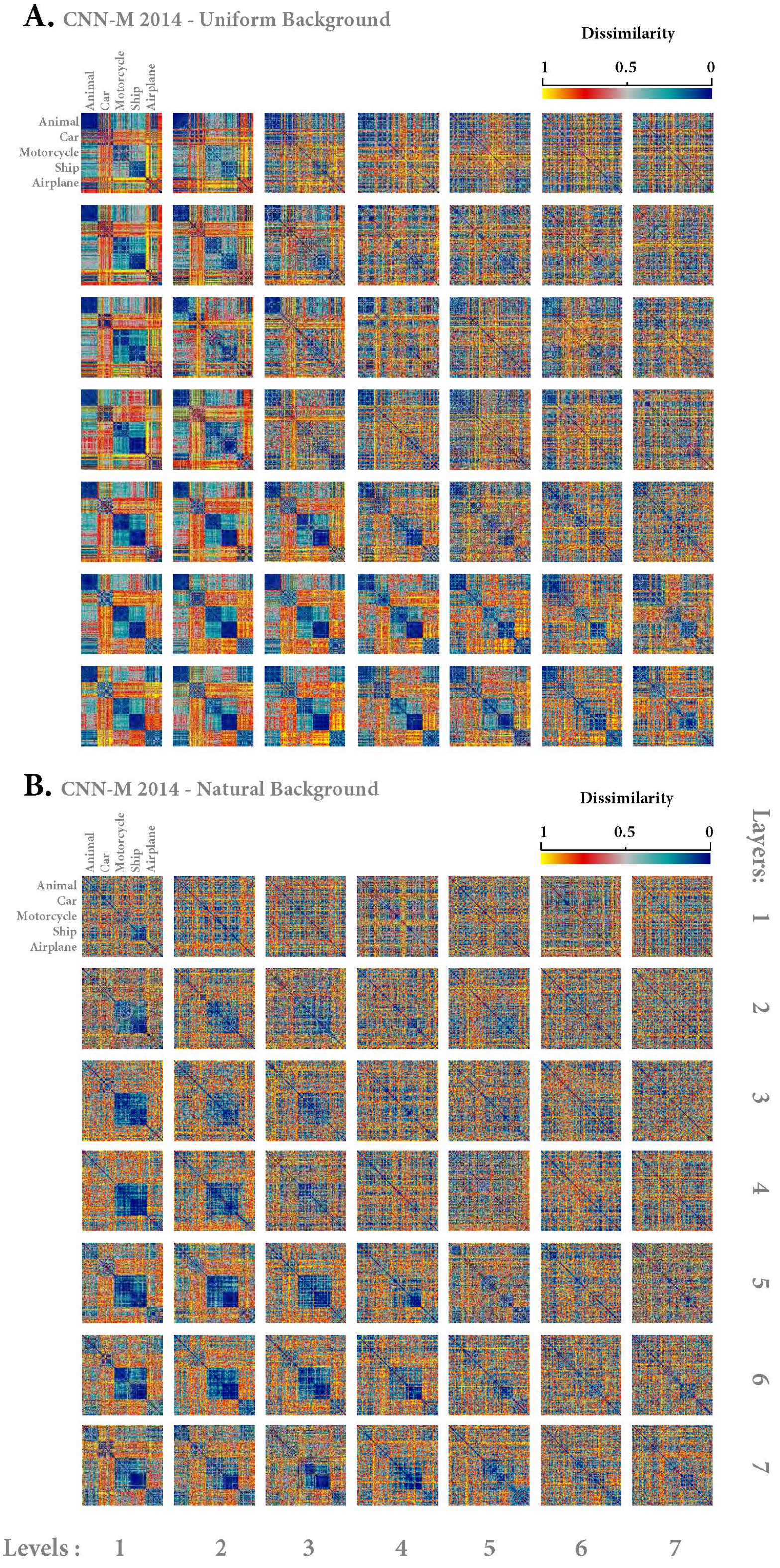

Figure S13. Representational Dissimilarity Matrices (RDM) for different layers of CNN-M 2014 in A) plain and B) natural backgrounds. Each element in a matrix shows the pairwise dissimilarities between the internal representations of a model for two images (measured as 1-r, Spearman's rank correlation. See Materials and Methods). Each row of RDMs corresponds to a layer and each column shows a particular level of variation (from level 1-7). The color bar at the top-right corner shows the degree of dissimilarity. The size of each matrix is 100×100, with 20 randomly selected images from each category. This was done for the sake of better visualization.

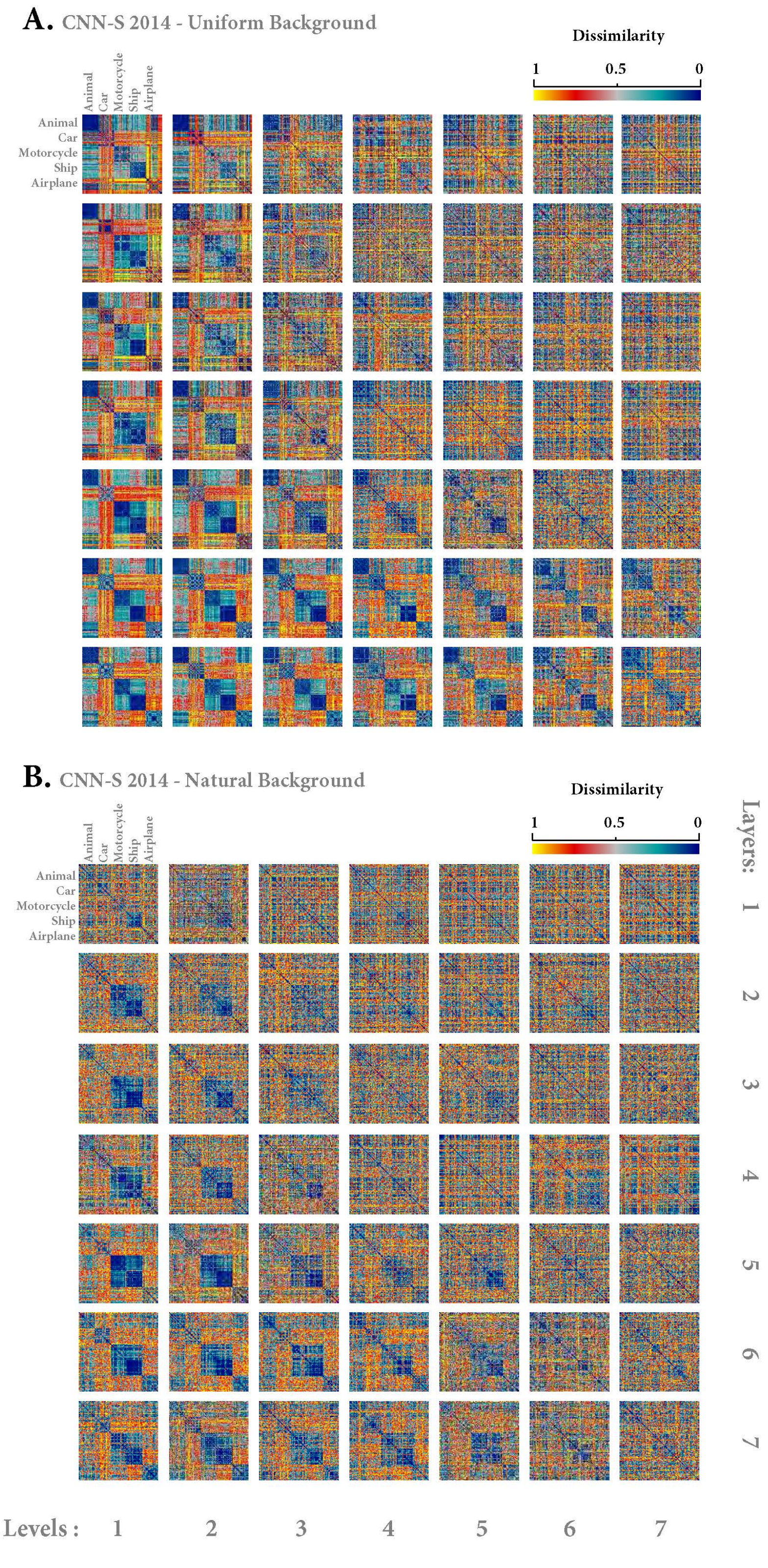

Figure S14. Representational Dissimilarity Matrices (RDM) for different layers of CNN-S 2014 in A) plain and B) natural backgrounds. Each element in a matrix shows the pairwise dissimilarities between the internal representations of a model for two images (measured as 1-r, Spearman's rank correlation. See Materials and Methods). Each row of RDMs corresponds to a layer and each column shows a particular level of variation (from level 1-7). The color bar at the top-right corner shows the degree of dissimilarity. The size of each matrix is 100×100, with 20 randomly selected images from each category. This was done for the sake of better visualization.

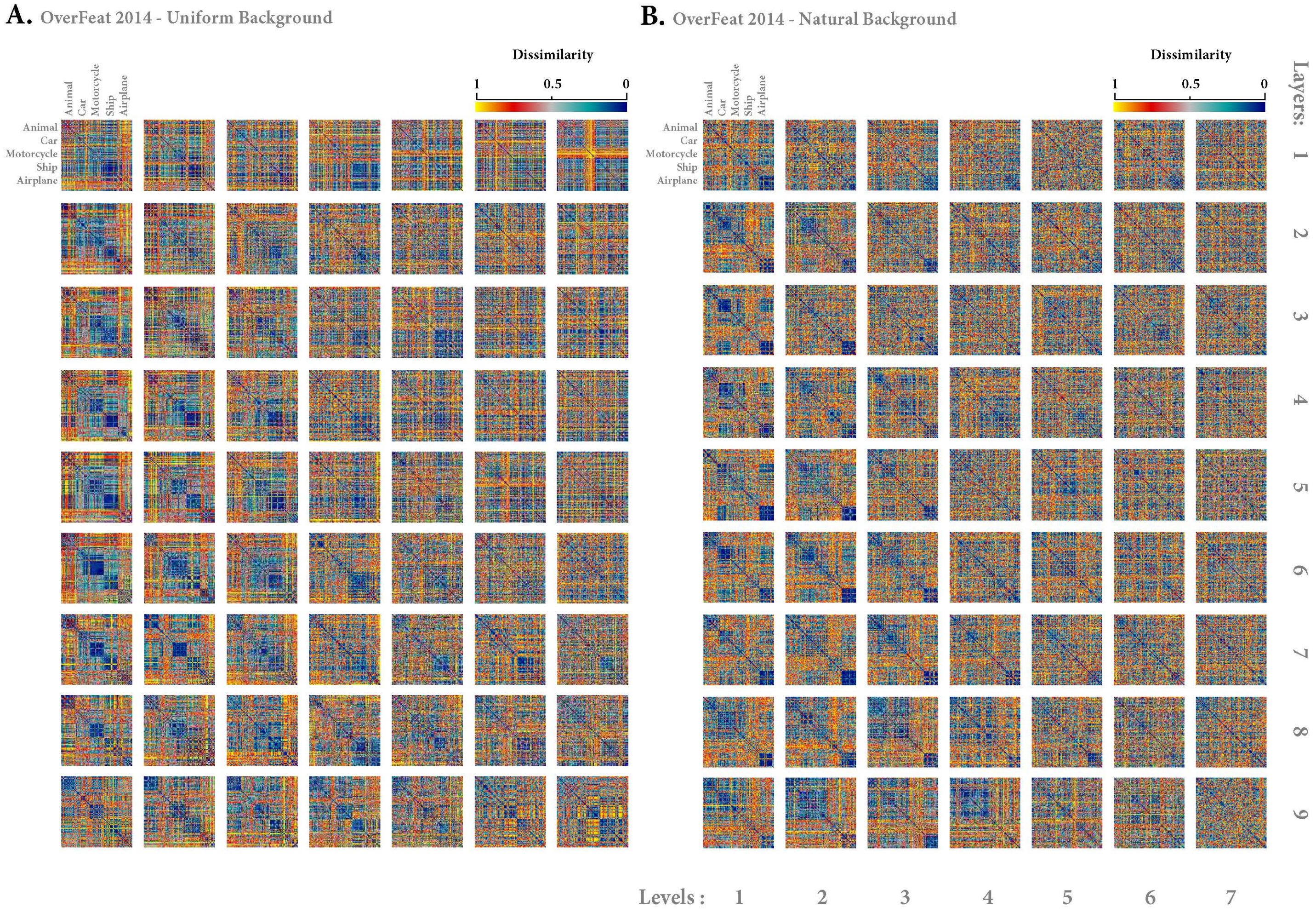

Figure S15. Representational Dissimilarity Matrices (RDM) for different layers of OverFeat 2014 in A) plain and B) natural backgrounds. Each element in a matrix shows the pairwise dissimilarities between the internal representations of a model for two images (measured as 1-r, Spearman's rank correlation. See Materials and Methods). Each row of RDMs corresponds to a layer and each column shows a particular level of variation (from level 1-7). The color bar at the top-right corner shows the degree of dissimilarity. The size of each matrix is 100×100, with 20 randomly selected images from each category. This was done for the sake of better visualization.

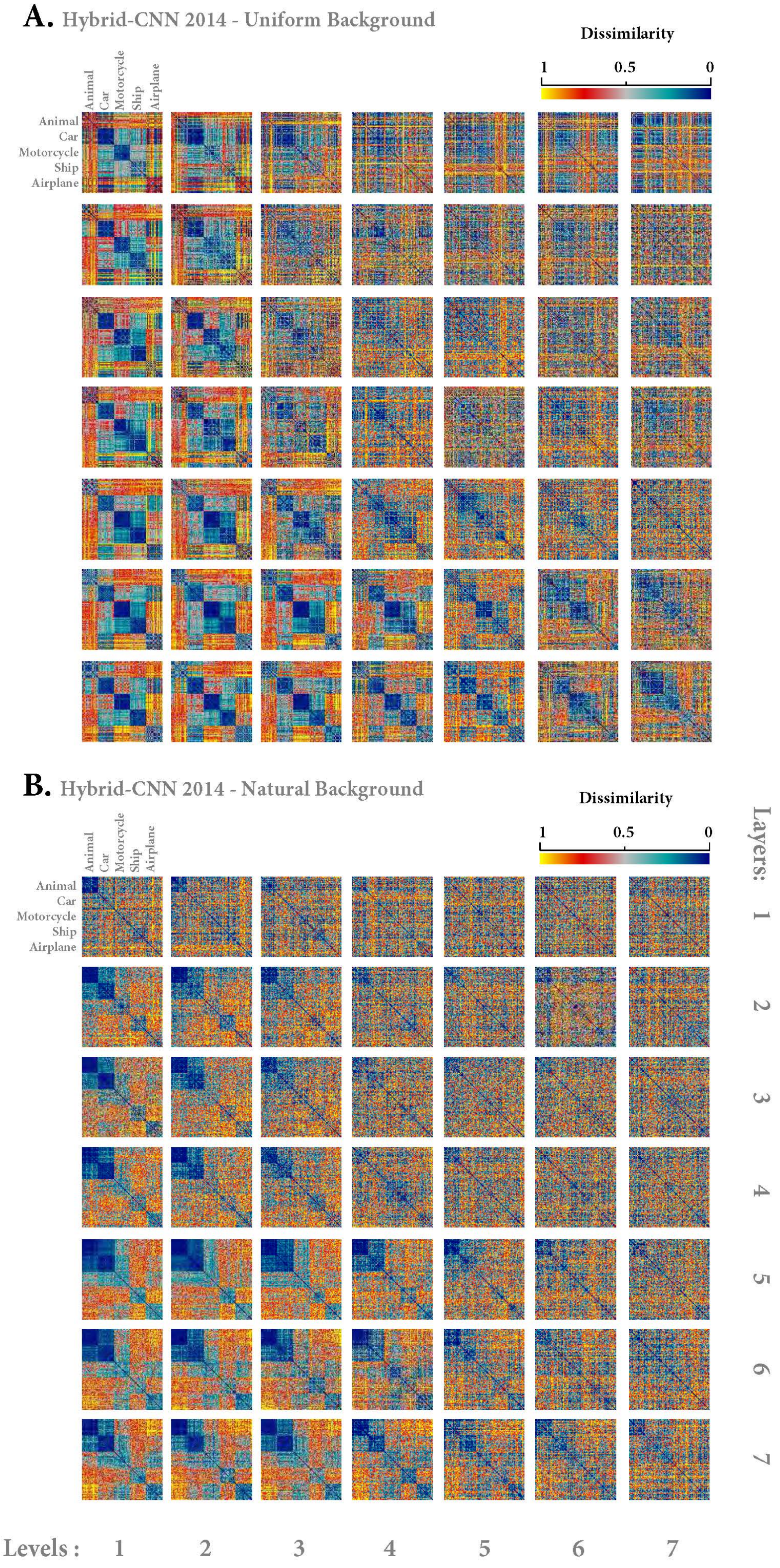

Figure S16. Representational Dissimilarity Matrices (RDM) for different layers of Hybrid-CNN 2014 in A) plain and B) natural backgrounds. Each element in a matrix shows the pairwise dissimilarities between the internal representations of a model for two images (measured as 1-r, Spearman's rank correlation. See Materials and Methods). Each row of RDMs corresponds to a layer and each column indicates a particular level of variation (from level 1-7). The color bar at the top-right corner shows the degree of dissimilarity. The size of each matrix is 100 × 100, with 20 randomly selected images from each category. This was done for the sake of better visualization.

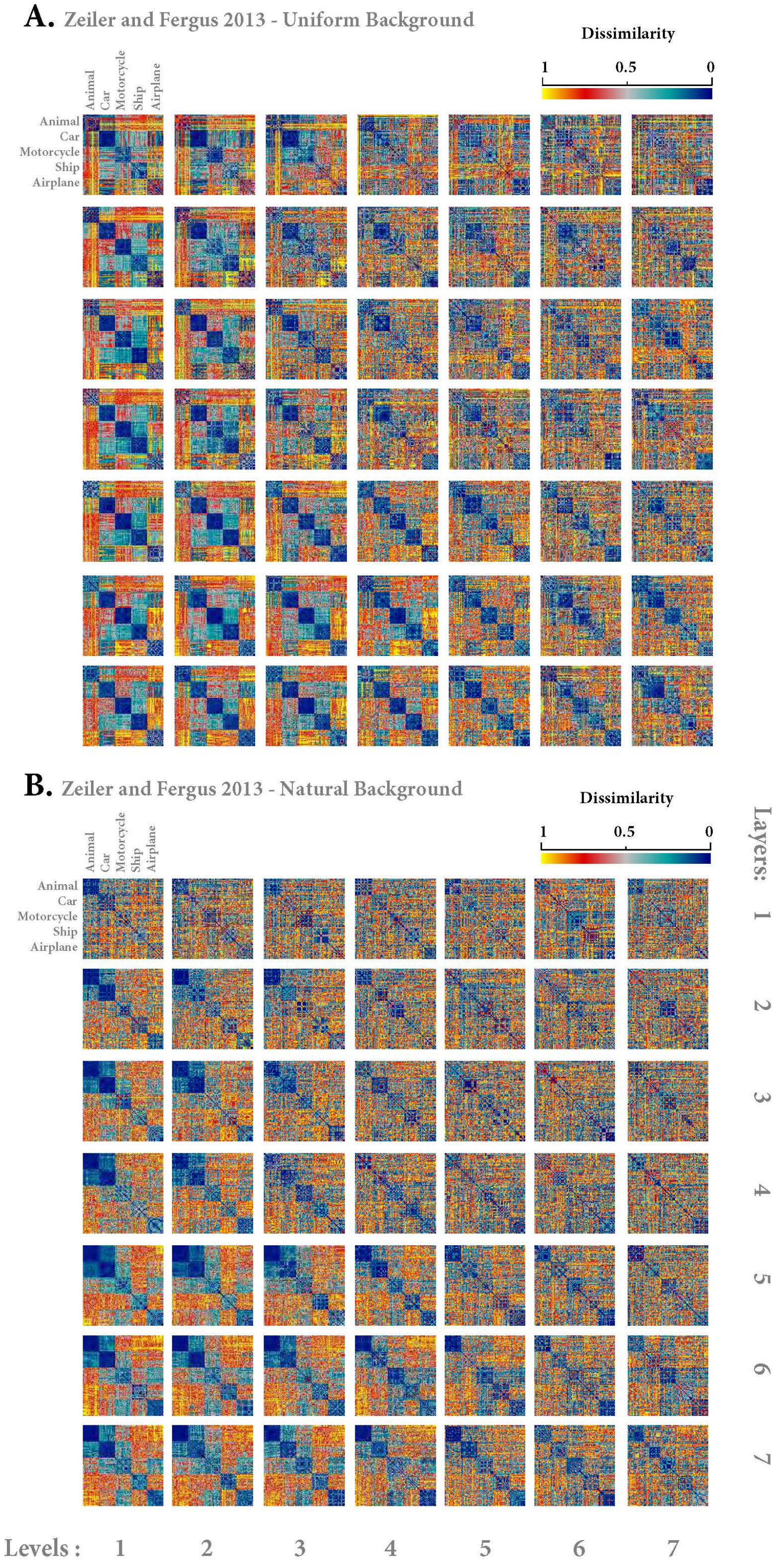

Figure S17. Representational Dissimilarity Matrices (RDM) for different layers of Zeiler and Fergus 2013 in A) plain and B) natural backgrounds. Each element in a matrix shows the pairwise dissimilarities between the internal representations of a model for two images (measured as 1-r, Spearman's rank correlation. See Materials and Methods). Each row of RDMs corresponds to a layer and each column shows a particular level of variation (from level 1-7). The color bar at the top-right corner shows the degree of dissimilarity. The size of each matrix is 100×100, with 20 randomly selected images from each category. This was done for the sake of better visualization.

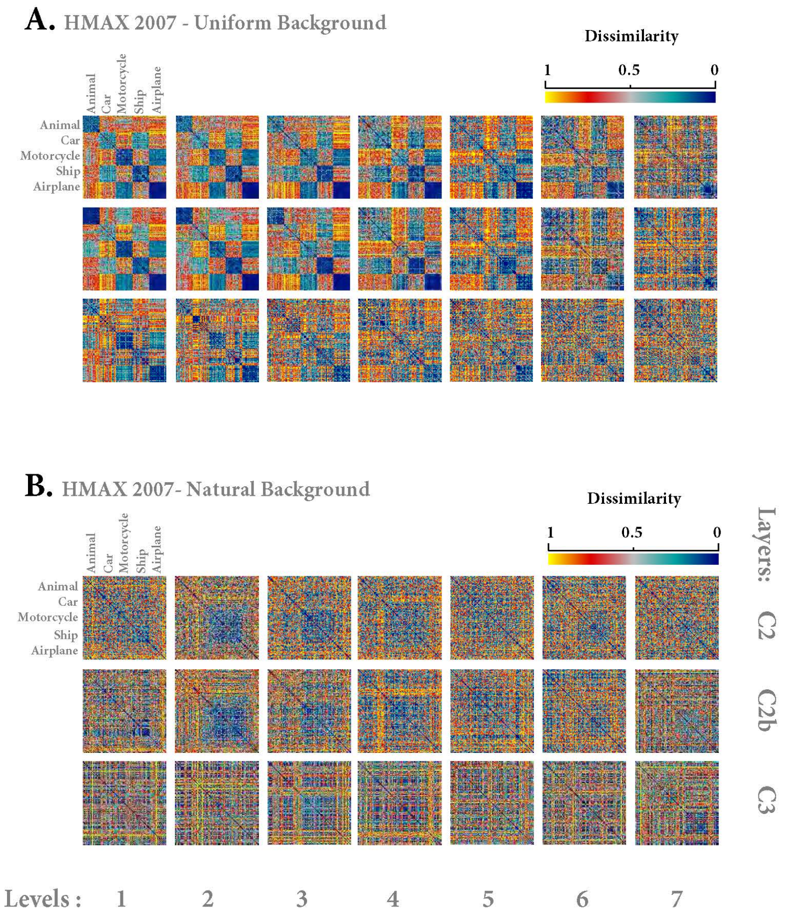

Figure S18. Representational Dissimilarity Matrices (RDM) for different layers of HMAX model in A) plain and B) natural backgrounds. Each element in a matrix shows the pairwise dissimilarities between the internal representations of a model for two images (measured as 1-r, Spearman's rank correlation. See Materials and Methods). Each row of RDMs corresponds to a layer and each column shows a particular level of variation (from level 1-7). The color bar at the top-right corner shows the degree of dissimilarity. The size of each matrix is 100×100, with 20 randomly selected images from each category. This was done for the sake of better visualization.

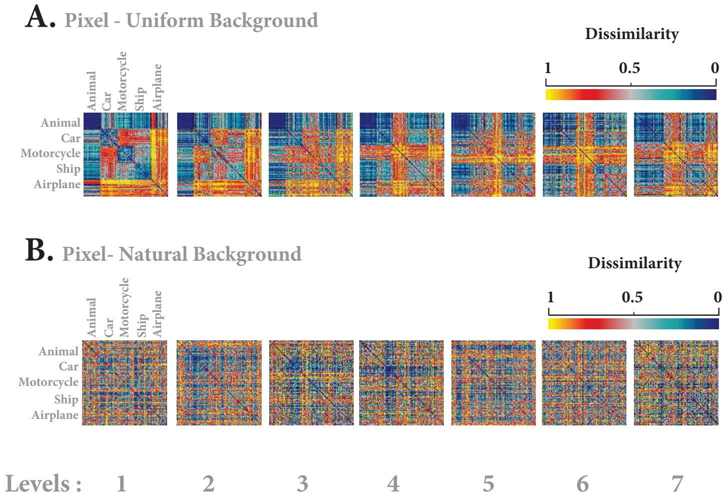

Figure S19. Representational Dissimilarity Matrices (RDM) of Pixel model for different levels of variation (from level 1-7) in A) plain and B) natural background conditions. Each element in a matrix shows the pairwise dissimilarities between the pixel-based representations of two images (measured as 1-r, Spearman's rank correlation. See Materials and Methods). The color bar at the top-right corner shows the degree of dissimilarity. The size of each matrix is 100×100, with 20 randomly selected images from each category. This was done for the sake of better visualization.

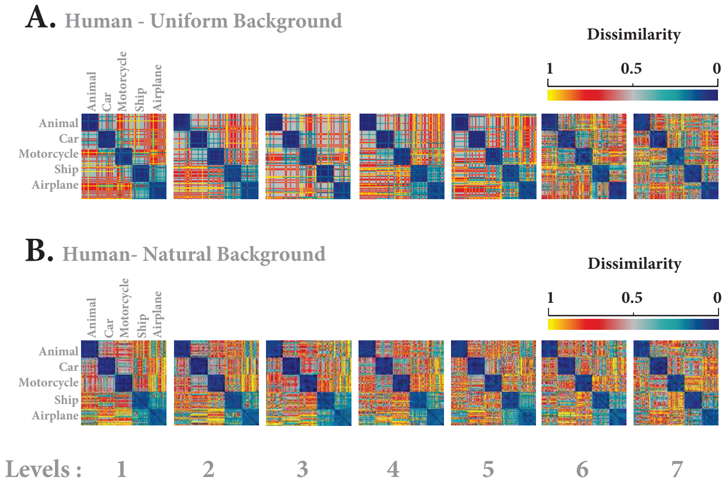

Figure S20. Representational Dissimilarity Matrices (RDM) of humans for different levels of variation (from level 1-7) in A) plain and B) natural background conditions. Each element in a matrix shows the pairwise dissimilarities between the approximated internal representations of humans for two images (measured as 1-r, Spearman's rank correlation. See Materials and Methods). The color bar at the top-right corner shows the degree of dissimilarity. The size of each matrix is 100×100, with 20 randomly selected images from each category. This was done for the sake of better visualization.

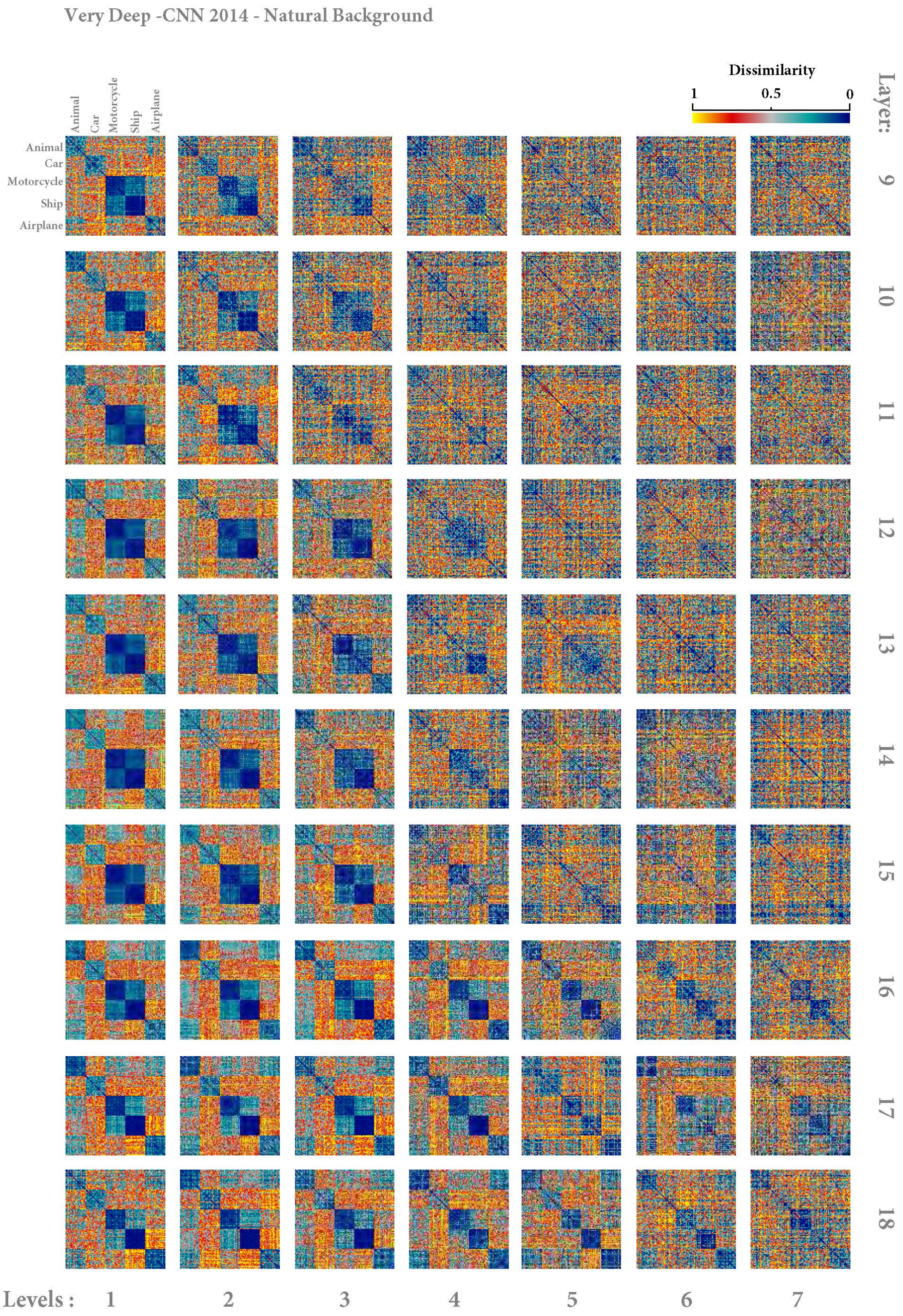

Figure S21. Representational Dissimilarity Matrices (RDM) of Very Deep model (layers 9 to 18) for different levels of variation (from level 1-7) in natural background condition. Each element in a matrix shows the pairwise dissimilarities between the representations of two images (measured as 1-r, Spearman's rank correlation. See Materials and Methods). The color bar at the top-right corner shows the degree of dissimilarity. The size of each matrix is 100×100, with 20 randomly selected images from each category. This was done for the sake of better visualization.

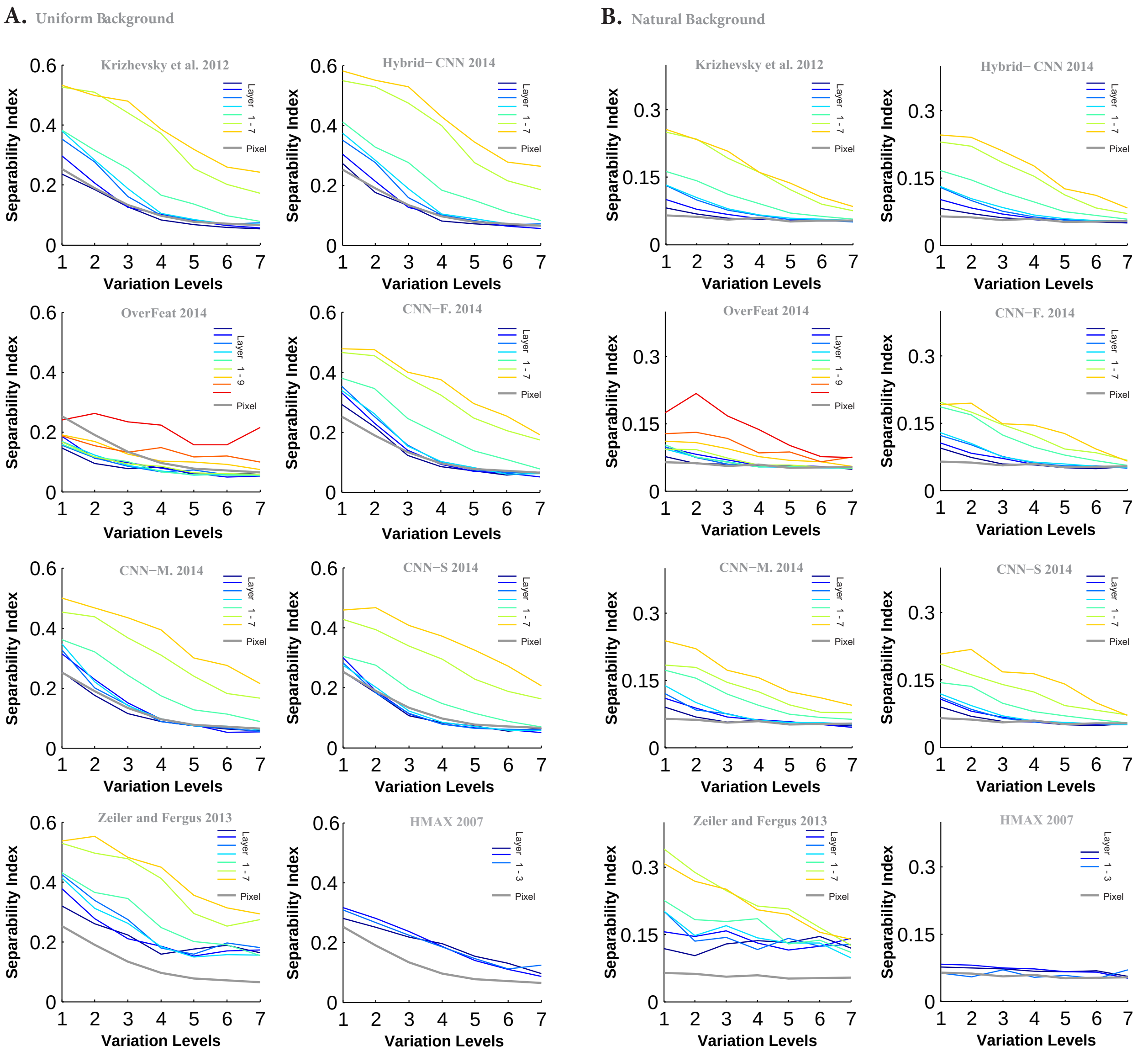

Figure S22. Separability index for DCNNs, HMAX and Pixel model. Panels in A show the separability index for the DCNNs when they fed with objects on uniform background and B shows the indexes for the case of natural backgrounds.
